# Evaluating Mediterranean diet and risk of chronic disease in cohort studies: an umbrella review of meta-analyses

**DOI:** 10.1007/s10654-018-0427-3

**Published:** 2018-07-20

**Authors:** Cecilia Galbete, Lukas Schwingshackl, Carolina Schwedhelm, Heiner Boeing, Matthias B. Schulze

**Affiliations:** 1 Department of Molecular Epidemiology, German Institute of Human Nutrition Potsdam-Rehbruecke, Nuthetal, Germany; 2 NutriAct – Competence Cluster Nutrition Research Berlin-Potsdam, Nuthetal, Germany; 3 Department of Epidemiology, German Institute of Human Nutrition Potsdam-Rehbruecke, Nuthetal, Germany; 4 Institute of Nutritional Sciences, University of Potsdam, Nuthetal, Germany

**Keywords:** Mediterranean diet, Chronic diseases, Umbrella review, Meta-analyses, Cohort studies, Heterogeneity

## Abstract

Several meta-analyses have been published summarizing the associations of the Mediterranean diet (MedDiet) with chronic diseases. We evaluated the quality and credibility of evidence from these meta-analyses as well as characterized the different indices used to define MedDiet and re-calculated the associations with the different indices identified. We conducted an umbrella review of meta-analyses on cohort studies evaluating the association of the MedDiet with type 2 diabetes, cardiovascular disease, cancer and cognitive-related diseases. We used the AMSTAR (A MeaSurement Tool to Assess systematic Reviews) checklist to evaluate the methodological quality of the meta-analyses, and the NutriGrade scoring system to evaluate the credibility of evidence. We also identified different indices used to define MedDiet; tests for subgroup differences were performed to compare the associations with the different indices when at least 2 studies were available for different definitions. Fourteen publications were identified and within them 27 meta-analyses which were based on 70 primary studies. Almost all meta-analyses reported inverse associations between MedDiet and risk of chronic disease, but the credibility of evidence was rated low to moderate. Moreover, substantial heterogeneity was observed on the use of the indices assessing adherence to the MedDiet, but two indices were the most used ones [Trichopoulou MedDiet (tMedDiet) and alternative MedDiet (aMedDiet)]. Overall, we observed little difference in risk associations comparing different MedDiet indices in the subgroup meta-analyses. Future prospective cohort studies are advised to use more homogenous definitions of the MedDiet to improve the comparability across meta-analyses.

## Introduction

The Seven Countries Study observed in the 1960s a lower cardiovascular mortality in the participating countries around the Mediterranean area [1]. Ancel Keys attributed this observation to the traditional diets such as the high olive oil consumption in Greece, the high fish intake in the Dalmatian area (Croatia), and the high vegetable intake in Italy. Later on, the dietary components considered to have a beneficial effect on health were combined in one index and published as MedDiet index [2]. Apart from the influence of the Mediterranean diet (MedDiet) on risk of cardiovascular disease (CVD), several metabolic diseases have been studied for a possible favourable role, including type 2 diabetes (T2D), cognitive-related diseases, and different types of cancer in the Mediterranean countries [3–10]. Recently the landmark PREDIMED study has been retracted and republished due to “irregularities in randomisation procedures” [11, 12]. Similarly to the retracted paper, the new findings showed that the incidence of combined cardiovascular events was lower among those assigned to a MedDiet supplemented with extra-virgin olive oil or nuts than among those assigned to a lower-fat diet [12].

The interest in the MedDiet as a whole was not restricted to those countries and thus, the MedDiet has been investigated in many countries that are geographically far from being Mediterranean, e.g. Australia and Japan [13–15]. The observed results have been summarised in several meta-analyses, but the methods used for the estimation of MedDiet adherence in the different study populations were often heterogeneous, implying the creation of many different indices intended to reflect MedDiet. In fact, even though most of the meta-analyses are consistent in their findings, the observed statistical heterogeneity within these varies from low to high (e.g. from 26% in the study from Jannasch et al. [16] on MedDiet and T2D to 82% in the meta-analysis on MedDiet and cancer from Schwingshackl et al. [17]). Moreover, the methodological quality and credibility of evidence of these meta-analyses have not been analysed so far.

The creation of nutritional indices is a common tool largely used in the nutritional epidemiology research with the purpose of reflecting the adherence to a dietary pattern, e.g. the MedDiet, in a certain population. These are created by ranking the population according to the intake of foods considered to be in line with, or against, the dietary pattern under study.

Sofi et al. as well as Davis et al. discussed the derived difficulties from the use of different scores measuring MedDiet adherence [18, 19]. They criticised that the use of different population-specific cut-off values for the consumption of the food groups considered to be part of the MedDiet hampered the further clinical and public health applications. But this is not the only discrepancy observed among the different scores; both authors mentioned also the inconsistencies observed in the classification of the food groups. However, this point has not been deeply investigated and could be one of the reasons for the high statistical heterogeneity found in several of the published meta-analyses. Thus, by the present complementary umbrella review, in a first step, we aimed to evaluate the methodological quality and the credibility of evidence generated by all available meta-analyses evaluating the association between adherence to a MedDiet and major chronic diseases (T2D, CVD, cancer and cognitive-related disorders) in cohort studies. In a second step, we aimed to enumerate and characterize the different definitions used to assess adherence to the MedDiet in the studies gathered in the meta-analyses, and to emulate the associations on chronic diseases with the different dietary MedDiet adherence indices.

## Methods

This manuscript was drafted in adherence to the recommendations of the ‘Preferred Reporting Items for Systematic reviews and Meta-Analyses’ (PRISMA) checklist [20].

The methodological approach of this umbrella review is based on previous published umbrella reviews, which focused on nut and garlic intake and risk of CVD [21, 22], and is next explained.

### Data sources and search strategy

PubMed (from inception until 27th March 2018) and Embase (from inception until 27th March 2018) was searched for meta-analyses published in English language using following search terms: (Mediterranean[tiab]) AND (cardiovascular[tiab] OR coronary[tiab] OR myocardial[tiab] OR stroke[tiab] OR mortality[tiab] OR cancer[tiab] OR “neoplastic disease”[tiab] OR tumor[tiab] OR diabetes[tiab] OR “cognitive decline”[tiab] OR cognition[tiab] OR dementia[tiab] OR Alzheimer disease[tiab]) AND (meta-analysis[tiab]). Additionally, reference lists of the included meta-analyses were verified for further relevant studies as well.

### Inclusion criteria

Studies were included in this umbrella review if they met all of the following criteria: (1) meta-analysis of cohort studies, (2) evaluating the association of scores used for assessing adherence to a MedDiet or considered by the authors as reflecting a MedDiet type diet, (3) study population: ≥ 18 years, (4) study endpoints include overall cancer mortality and/or incidence, CVD or coronary heart disease (CHD) mortality and/or incidence, stroke, myocardial infarction (MI), acute myocardial infarction (AMI) type 2 diabetes, Alzheimer’s disease (AD), mild cognitive impairment (MCI), or dementia.

### Methodological quality

The methodological quality was evaluated using a modified version of the AMSTAR (A MeaSurement Tool to Assess systematic Reviews) [23] checklist, which has been recently established to evaluate the methodological quality of meta-analyses and systematic reviews on the Mediterranean diet and CVD outcomes and is based on 14 items (maximum score of 22) [24]. These are grouped within four different domains; (1) “a priori design”, which includes two questions, (2) Literatures search and duplicate effort, which includes five different questions, (3) coding of studies, including two questions, and (4) analysis and interpretation, which includes four different questions. The original AMSTAR checklist has been previously used to choose high quality systematic reviews and meta-analysis to build the Dietary Guidelines for Americans 2015–2020 [25].

### Credibility of the evidence

To evaluate credibility of evidence for the association between adherence to MedDiet and risk of the included outcomes we applied the NutriGrade scoring system (max 10 points) which comprises the following items for cohort studies [26]: (1) risk of bias/study quality/study limitations, (2) precision, (3) heterogeneity, (4) directness, (5) publication bias, (6) funding bias, (7) effect size, and (8) dose–response. Based on this scoring system we recommend four categories to judge credibility of meta-evidence: high, moderate, low, and very low taking into account the following cut-points: ≥ 8 points (high meta-evidence), 6–7.99 points (moderate meta-evidence), 4–5.99 (low meta-evidence), and 0–3.99 (very low meta-evidence).

### Identification of different MedDiet scores

Within the meta-analyses included in this study the different scores used in the primary studies were carefully evaluated. The nine food groups referred to by Trichopoulou et al. [27], and usually quoted for the creation of the MedDiet score were considered as the basis (fruit, vegetables, legumes, cereals, meat, dairy products, fish, alcohol and, healthy fats), and for each primary study we carefully identified the foods reported to be included within the different food groups. Thus, the scores were considered to be different when: (1) different food groups were included, (2) food groups were comprised of different food items, and (3) different specific cut-off values within the food items were applied. This last point was particularly relevant for the different cut-off values used to define “moderate alcohol intake”.

### Statistical analysis

If at least two cohort studies were included in a meta-analysis for at least two specific MedDiet adherence scores identified (e.g. tMedDiet, aMedDiet, sMedDiet) a new meta-analysis was carried out to estimate and compare the associations between these scores for the included outcomes. These new meta-analyses were performed by combining the multivariable adjusted RRs, HR of the highest compared with the lowest MedDiet adherence category, or 2-point increase in MedDiet adherence score based on a random effects model using the DerSimonian–Laird method, which incorporated both within and between study variability [28]. To evaluate the weighting of each study, the standard error for the logarithm HR/RR/OR of each study was calculated and regarded as the estimated variance of the logarithm HR/RR/OR, using an inverse variance method [28].

To detect discrepancies between the different types of MedDiet adherence scores for an outcome a test for subgroup differences was performed based on a random effects model (fixed effects model used for the sensitivity analysis).

For the summary random effects, we estimated for each meta-analysis the 95% prediction interval (PI), which further accounts for the degree of between-study heterogeneity and gives a range for which we are 95% confident that the effect in a new study examining the same association lies within [29].

All analyses were conducted using the Review Manager by the Cochrane Collaboration (version 5.3) and Stata 14.2 (Stata-Corp, College Station, TX. USA).

## Results

The study characteristics of the meta-analyses are summarized in Table 1. A total of 14 publications reporting 27 meta-analyses on the association of the MedDiet with the risk of any of the major chronic diseases (T2D, CVD, cancer, or cognitive-related diseases) were included in the present umbrella review [16, 17, 19, 30–40]. Among all the meta-analyses 70 primary studies were included [2, 7, 13, 27, 41–106]. Several different endpoints have been evaluated: T2D, CVD incidence and/or mortality, CHD incidence, different types of stroke incidence and/or mortality, MI incidence, MCI incidence, AD incidence, and dementia incidence. Four meta-analyses were found evaluating the association between adherence to the MedDiet and risk of T2D [16, 30, 31, 40], among which 11 primary studies were considered [7, 43, 46, 50, 61, 74, 76, 79, 82, 83, 98]. Comparing the highest versus lowest adherence category an inverse association between 13% (RR 0.87, 95% CI 0.82, 0.97) and 23% (RR 0.77, 95% CI 0.66, 0.89) for the risk of T2D was observed. Regarding the different CVD endpoints a total of 12 meta-analyses within 5 publications were identified [19, 32–35], and within these 31 primary studies were included [2, 13, 41, 44, 47, 49, 51, 52, 56, 59, 60, 67, 71, 72, 75, 77, 78, 80, 85–87, 89–93, 96, 100, 103–105]. A 2-point increase in adherence to the MedDiet score was associated with a 10% (RR 0.90, 95% CI 0.86, 0.94) lower risk of CVD incidence/mortality [19, 34]. Comparing the highest versus lowest category of adherence to the MedDiet the risk of CVD was reduced by approximately 19% (RR 0.81, 95% CI 0.74, 0.88) to 27% (RR 0.73, 95% CI 0.66, 0.80). Similar results were reported for CHD (RR 0.72, 95% CI 0.60, 0.86), MI (RR 0.67, 95% CI 0.54, 0.83), AMI (RR 0.74, 95% CI 0.66, 0.83), and stroke (RR 0.77, 95% CI 0.67, 0.90). No association was observed between adherence to the MedDiet and haemorrhagic stroke. Regarding overall cancer incidence and/or mortality four meta-analyses were identified within three publications [17, 19, 36], and within these a total of 27 primary studies were identified [27, 42, 45, 48, 53–55, 57–59, 62, 68–70, 72, 73, 75, 78, 80, 84, 94, 96, 97, 99–102]. Two meta-analyses considering overall cancer mortality and one meta-analysis including overall cancer incidence were found [17, 36], whereas in another meta-analysis conducted by Sofi et al. [19] cancer mortality and incidence were combined. Comparing the highest versus lowest adherence to MedDiet category a 14% (RR 0.86, 95% CI 0.81, 0.91) (4% reduction for cancer incidence) reduced risk of cancer mortality was reported, whereas a 2-point increase in the MedDiet score was associated with a 5% (RR 0.95 95% CI 0.93, 0.97) reduced risk of cancer mortality/incidence. Regarding cognitive-related disorders three studies meta-analysed the observed effects of MedDiet with different outcomes: MCI, AD and dementia [37–39]. A total of 6 meta-analyses were conducted [37–39]. Comparing highest versus lowest category of adherence to the MedDiet up to 31% (RR 0.69, 95% CI 0.57, 0.84) risk reduction for MCI was observed and 40% (RR 0.60, 95% CI 0.48, 0.77) reduction for AD. No association was observed for incident dementia.

**Table 1:** General and specific characteristics of the included meta-analysis

References	Outcome and summary of the estimates RR (95% CI)	No. studies included	Sample size, cases	Follow-up (years)	Predictive Interval (95% CI)	NutriGrade grading [26]	Modified AMSTAR rating [24]	Included MedDiet-scores* (ref.)	Results re-analyses RR (95% CI) and test for subgroup differences (random effects model)	Results re-analyses RR (95% CI) and test for subgroup differences (fixed effects model)
Jannasch et al. [16]	T2D incidence RR: 0.87 (0.82, 0.97) I^2^ = 26%	6 cohort studies	183,392, 17,561	10–20	(0.77, 1.00)	Low	18/22	tMedDiet: [7, 50] tMedDiet 4: [61] aMedDiet: [74] aMedDiet 2: [79] aMedDiet 4: [82]	High versus low adherence RR (tMedDiet): 0.87 (0.81, 0.93) RR (aMedDiet): 0.88 (0.75, 1.04) Test for subgroup differences (tMedDiet vs aMedDiet): *p* = 0.83	High versus low adherence RR (tMedDiet): 0.87 (0.81, 0.93) RR (aMedDiet): 0.89 (0.84, 0.98) Test for subgroup differences (tMedDiet vs aMedDiet): *p* = 0.51
Schwingshackl et al. [40]	T2D incidence RR: 0.83 (0.74, 0.92) I^2^ = 56%	8 cohort studies	129,647, 19,463	3.2–20	(0.61, 1.11)	Moderate	17/22	tMedDiet: [46, 50] tMedDiet 4: [61] aMedDiet: [76] aMedDiet 2: [79] aMedDiet 4: [82] CA: [98] MedDiet 1: [83]	High versus low adherence RR (tMedDiet): 0.86 (0.74, 1.01) RR (aMedDiet): 0.82 (0.68, 1.00) Test for subgroup differences (tMedDiet vs aMedDiet): *p* = 0.70	High versus low adherence RR (tMedDiet): 0.88 (0.81, 0.95) RR (aMedDiet): 0.79 (0.71, 0.87) Test for subgroup differences (tMedDiet vs aMedDiet): *p* = 0.11
Koloverou et al. [30]	T2D incidence RR: 0.77 (0.66, 0.89) I^2^ = 58%	9 cohort studies	135,168, 19,609	3.2–20	(0.62, 1.15)	Low	16/22	tMedDiet: [43, 46, 50] tMedDiet 4: [61] aMedDiet: [76] aMedDiet 2: [79] aMedDiet 4: [82] CA: [98] MedDiet 1: [83]	High versus low adherence RR (tMedDiet): 0.88 (0.76, 1.02) RR (aMedDiet): 0.83 (0.67, 1.04) Test for subgroup differences (tMedDiet vs aMedDiet): *p* = 0.66	High versus low adherence RR (tMedDiet): 0.88 (0.81, 0.95) RR (aMedDiet): 0.79 (0.71, 0.89) Test for subgroup differences (tMedDiet vs aMedDiet): *p* = 0.12
Esposito et al. [31]	T2D incidence RR: 0.80 (0.68, 0.93) I^2^ = 64%	6 cohort studies	95,384, 7129	3.2–20	(0.50, 1.26)	Low	17/22	MedDiet 1: [83] tMedDiet: [46, 50] aMedDiet 2: [79] aMedDiet: [76] aMedDiet 4: [82]	High versus low adherence RR (tMedDiet): 0.46 (0.09, 2.21) RR (aMedDiet): 0.82 (0.68, 1.00) Test for subgroup differences (tMedDiet vs aMedDiet): *p* = 0.47	High versus low adherence RR (tMedDiet): 0.87 (0.77, 0.98) RR (aMedDiet): 0.79 (0.71, 0.87) Test for subgroup differences (tMedDiet vs aMedDiet): *p* = 0.21
Rosato et al. [32]	CHD/AMI incidence/mortality RR: 0.74 (0.66, 0.83) I^2^ = 27%	9 cohort studies	392,283, 4256	4.9–20	(0.50, 0.99)	Moderate	14/22	MedDiet 6: [90] tMedDiet 4: [60] tMedDiet: [44, 47] tMedDiet 2: [56] MedDiet 3: [85] mMedDiet: [96] aMedDiet: [77] MedDiet 7: [91]	NA	NA
	Unspecified stroke incidence/mortality RR: 0.77 (0.67, 0.90) I^2^ = 0%	5 cohort studies	107,074, 1210	No follow-up to 20	(0.61, 0.98)	Low		tMedDiet: [41, 49, 51] aMedDiet: [77] MedDiet 3: [85] MedDiet 8: [93]	NA	NA
	Ischemic stroke RR: 0.82 (0.73, 0.92) I^2^ = 0%	5 cohort studies	181,295, 2997	6.5–20	(0.67, 1.00)	Low		tMedDiet: [41] tMedDiet 2: [56] tMedDiet 8: [67] aMedDiet: [71] MedDiet 7: [91]	NA	NA
	Haemorrhagic stroke RR: 1.01 (0.74, 1.37) I^2^ = 36%	4 cohort studies	178,727, 737	6.5–20	(0.36, 2.82)	Low		tMedDiet: [41] tMedDiet 8: [67] aMedDiet: [71] MedDiet 7: [91]	NA	NA
	Unspecified CVD incidence/mortality RR: 0.81 (0.74, 0.88) I^2^ = 80%	11 cohort studies	831,642, 56 695	4.9–20	(0.62, 1.06)	Moderate		tMedDiet: [47, 52] tMedDiet 2: [56] tMedDiet 4: [59] aMedDiet: [72, 75, 77] MedDiet 3: [85] MedDiet 4: [87] MedDiet 7: [92] mMedDiet: [96]	High versus low adherence RR (tMedDiet): 0.69 (0.54, 0.89) RR (aMedDiet): 0.83 (0.74, 0.94) Test for subgroup differences (tMedDiet vs aMedDiet): *p* = 0.18	High versus low adherence RR (tMedDiet): 0.70 (0.58, 0.86) RR (aMedDiet): 0.81 (0.79, 0.84) Test for subgroup differences (tMedDiet vs aMedDiet): *p* = 0.17
Grosso et al. [33]	CVD incidence RR: 0.73 (0.66, 0.80) I^2^ = 36%	13 cohort studies	274,023, 11,688	2–20	(0.57, 0.92)	Moderate	16/22	tMedDiet: [44, 47, 49] tMedDiet 2: [56] tMedDiet 4: [60] aMedDiet: [71] iMedDiet: [41] MedDiet 5: [89] MedDiet 3: [85, 86] MAI: [105] sMedDiet: [103, 104]	High versus low adherence RR (tMedDiet): 0.71 (0.60, 0.85) RR (MedDiet 3): 0.80 (0.66, 0.97) RR (sMedDiet): 0.22 (0.06, 0.82) Test for subgroup differences (tMedDiet vs MedDiet 3 vs sMedDiet): *p* = 0.13	High versus low adherence RR (tMedDiet): 0.72 (0.63, 0.82) RR (MedDiet 3): 0.83 (0.74, 0.92) RR (sMedDiet): 0.21 (0.12, 0.37) Test for subgroup differences (tMedDiet vs MedDiet 3 vs sMedDiet): *p* < 0.01
	CVD mortality RR: 0.75 (0.68, 0.83) I^2^ = 75%	14 cohort studies	786,279, 9563	5.8–40	(0.53, 1.06)	Low		tMedDiet: [44, 49] tMedDiet 4: [13, 59] aMedDiet: [71] aMedDiet 1: [78] aMedDiet 3: [80] MAI: [105] MedDiet 3: [85] MedDiet 5: [89] rmMedDiet: [100] MedDiet 6: [90] mMedDiet: [96]	RR (tMedDiet): 0.76 (0.68, 0.85) RR (aMedDiet): 0.77 (0.68, 0.86) Test for subgroup differences (tMedDiet vs aMedDiet): *p* = 0.93	RR (tMedDiet): 0.76 (0.68, 0.85) RR (aMedDiet): 0.77 (0.71, 0.84) Test for subgroup differences (tMedDiet vs aMedDiet): *p* = 0.93
	CHD incidence RR: 0.72 (0.60, 0.86) I^2^ = NA	4 cohort studies	153,502, 2910	4.9–20	(0.38, 1.36)	Low		tMedDiet: [44, 47] tMedDiet 4: [60] aMedDiet: [71]	NA	NA
	MI incidence RR: 0.67 (0.54, 0.83) I^2^ = NA	3 cohort studies	44,428, 1364	9–14	(0.17, 2.70)	Low		tMedDiet 2: [56] MedDiet 3: [85] MedDiet 5: [89]	NA	NA
	Stroke incidence RR: 0.76 (0.60, 0.96) I^2^ = 52%	5 cohort studies	159,995, 2444	7.9–20	(0.37, 1.56)	Low		tMedDiet 2: [56] aMedDiet: [71] MedDiet 3: [85] MedDiet 5: [89] iMedDiet: [41]	NA	NA
Martinez-Gonzalez and Bes-Rastrollo [34]	CVD incidence/mortality RR: 0.90 (0.86, 0.94) I^2^ = 78%	12 cohort studies	671,005, 15,909	4.9–40	(0.79, 1.03)	Low	11/22	tMedDiet: [44, 47, 49] tMedDiet 2: [56] tMedDiet 4: [60] aMedDiet: [71] aMedDiet 1: [78] mMedDiet: [96] rmMedDiet: [100] MAI: [105] MedDiet 5: [89] MedDiet 3: [85]	Per 2-point increase RR(tMedDiet): 0.87 (0.81, 0.93) RR(aMedDiet): 0.91 (0.88, 0.95) Test for subgroup differences (tMedDiet vs aMedDiet): *p* = 0.22	Per 2-point increase RR(tMedDiet): 0.87 (0.82, 0.92) RR(aMedDiet): 0.91 (0.89, 0.94) Test for subgroup differences (tMedDiet vs aMedDiet): *p* = 0.14
Sofi et al. [19]	CVD incidence/mortality RR: 0.90 (0.87, 0.92) I^2^ = 38%	14 cohort studies	752,293, 16,631	4.5–20	(0.83, 0.97)	Moderate	14/22	MedDiet: [2] tMedDiet: [44, 47, 49] tMedDiet 2: [56] tMedDiet 4: [59, 60] aMedDiet: [71] aMedDiet 1: [78] MedDiet 3: [85] MedDiet 4: [87] iMedDiet: [41] mMedDiet: [96] rmMedDiet: [100]	Per 2-point increase RR (tMedDiet): 0.87 (0.83, 0.91) RR (aMedDiet): 0.92 (0.89, 0.94) Test for subgroup differences (tMedDiet vs aMedDiet): *p* = 0.08	Per 2-point increase RR (tMedDiet): 0.87 (0.83, 0.91) RR (aMedDiet): 0.92 (0.89, 0.94) Test for subgroup differences (tMedDiet vs aMedDiet): *p* = 0.06
	Cancer incidence/mortality RR: 0.95 (0.93, 0.97) I^2^ = 65%	14 cohort studies	2,720,221, 83,111	7.9–16	(0.88, 1.02)	Moderate		tMedDiet: [42, 45] tMedDiet 1: [53–55] tMedDiet 4: [58, 59] tMedDiet 5: [62] aMedDiet: [69, 70] aMedDiet 1: [78] mMedDiet: [96] rmMedDiet: [100] iMedDiet: [97]	Per 2-point increase RR (tMedDiet): 0.96 (0.94, 0.99) RR (aMedDiet): 0.93 (0.93, 0.98) Test for subgroup differences (tMedDiet vs aMedDiet): *p* = 0.76	Per 2-point increase RR (tMedDiet): 0.96 (0.96, 0.97) RR (aMedDiet): 0.95 (0.93, 0.98) Test for subgroup differences (tMedDiet vs aMedDiet): *p* = 0.42
Psaltopoulou et al. [35]	Stroke RR: 0.84 (0.74, 0.95) I^2^ = 0%	4 cohort studies	152,843, 2560	7.9–20	(0.68, 1.03)	Low	19/22	tMedDiet: [41] tMedDiet 2: [56] aMedDiet: [71] MedDiet 3: [85]	NA	NA
Schwingshackl et al. [17]	Cancer mortality RR: 0.86 (0.81–0.91) I^2^ = 82%	14 cohort studies	1,363,136, 54,569	6.3–40	(0.70, 1.05)	Moderate	18/22	tMedDiet: [45, 48] tMedDiet 3: [57] tMedDiet 9: [68] aMedDiet: [72, 73, 75] aMedDiet 3: [80] mMedDiet: [96] rmMedDiet: [100] MedDiet 2: [84] MedDiet 9: [94] FA: [99]	High versus low adherence RR (tMedDiet): 0.92 (0.74, 1.15) RR (aMedDiet): 0.80 (0.78, 0.83) Test for subgroup differences (tMedDiet vs aMedDiet): *p* = 0.22	High versus low adherence RR (tMedDiet): 0.83 (0.78, 0.88) RR (aMedDiet): 0.80 (0.78, 0.83) Test for subgroup differences (tMedDiet vs aMedDiet): *p* = 0.31
Bloomfield et al. [36]	Cancer mortality RR: 0.86 (0.82, 0.91) I^2^ = 77%	13 cohort studies	991,306, 49,819	5.8–40	(0.72, 1.04)	Low	15/22	tMedDiet: [27, 45] tMedDiet 3: [57] aMedDiet: [72, 73, 75] aMedDiet 3: [80] mMedDiet: [96] FA: [99] rmMedDiet: [100] MedDiet 2: [84] AHEI: [102]	High versus low adherence RR (tMedDiet): 0.92 (0.64, 1.31) RR (aMedDiet): 0.81 (0.78, 0.85) Test for subgroup differences (tMedDiet vs aMedDiet): *p* = 0.48	High versus low adherence RR (tMedDiet): 0.84 (0.69, 1.02) RR (aMedDiet): 0.81 (0.78, 0.83) Test for subgroup differences (tMedDiet vs aMedDiet): *p* = 0.68
	Cancer incidence RR: 0.96 (0.95, 0.97) I^2^ = 0%	3 cohort studies	591, 002, 48,683	8.7–24	(0.87, 1.05)	Low		tMedDiet 1: [53] AHEI: [101]	NA	NA
Wu and Sun [37]	MCI incidence RR: 0.83 (0.75, 0.93) I^2^ = 0%	5 cohort studies	27,667, 2376	2.2–12	(0.70, 1.00)	Moderate	18/22	tMedDiet 6: [63, 64] tMedDiet 8: [106] aMedDiet 3: [81] MedDiet 4: [88]	NA	NA
	AD incidence RR: 0.60 (0.48, 0.77) I^2^ = 0%	5 cohort studies	7609, 838	4–12	(0.41, 0.89)	Moderate		tMedDiet 6: [64, 65] tMedDiet 7: [66] MedDiet4: [88] MedDiet 10: [95]	NA	NA
	Dementia RR: 1.07 (0.81,1.42) I^2^ = 0%	3 cohort studies	9811, 632	5–12	(0.17, 6.61)	Low		tMedDiet 7: [66] aMedDiet 3: [81] MedDiet 4: [88]	NA	NA
Cao et al. [38]	MCI incidence RR: 0.69 (0.57, 0.84) I^2^ = 1%	4 cohort studies	766, 6294	2.2–5	(0.49, 0.96)	Low	10/22	tMedDiet 6: [63–65] tMedDiet 7: [66]	NA	NA
Singh et al. [39]	MCI incidence RR: 0.73 (0.56, 0.96) I^2^ = 0%	2 cohort studies	2626, 438	2.2–4.5	NA	Low	17/22	tMedDiet 6: [63, 64]	NA	NA
	AD incidence RR: 0.64 (0.46, 0.89) I^2^ = 0%	2 cohort studies	3668, 328	4–5	NA	Low		tMedDiet 7: [66] tMedDiet 6: [65]	NA	NA

*T2D* type 2 diabetes, *CVD* cardiovascular disease, *CHD* coronary heart disease, *MI* myocardial infarction, *AMI* acute myocardial infarction, *MCI* mild cognitive impairment, *AD* Alzheimer’s disease, *FA* factor analysis, *AHEI* Alternative Healthy Eating Index, *MAI* Mediterranean Adequacy Index, *CA* cluster analysis

*This makes reference to the indices identified in Table 2

Almost all included meta-analyses showed a significant inverse association between higher adherence to a MedDiet and risk of chronic diseases. Estimating 95% prediction intervals, however, the null value was excluded only in some of the associations (CVD incidence, CVD incidence/mortality, CHD/AMI incidence mortality, unspecified stroke incidence/mortality, AD incidence, and MCI incidence). This implies that most meta-analyses indicated high degrees of statistical heterogeneity and/or were based on a limited number of studies.

### Methodological quality

In total, the overall methodological quality of the included meta-analyses was rated as moderate (Table 1). On average, the meta-analyses achieved a mean of 16.5 points (75% of the maximum score).

### Credibility of the evidence

The NutriGrade credibility of evidence judgement varied between low (low confidence for the effect estimate: further research provides important information on the confidence and likely change the effect estimate) and moderate (moderate confidence for the effect estimate: further research could add information on the confidence and may change the effect estimate).

### Description of the different scores

A total of 70 primary studies were included within the 27 meta-analyses, where 34 different scores meant to reflect the MedDiet were applied [2, 7, 13, 27, 41–106]. A detailed description of the different definitions is shown in Table 2. Within the 34 different definitions gathered from the included studies, two main ones could be extracted; the first one made reference to the definitions derived from the one created in 2003 by Trichopoulou et al. [27] (tMedDiet), which, after the careful evaluation was considered as different from the first one used by Trichipoulou et al. [2]. The Trichopoulou definition from 2003 included nine food groups, five were postulated to be in line with it (vegetables, fruits and nuts, cereals, legumes, and fish), and two were in disagreement with it (dairy products and meat). Alcohol intake in moderation was also considered as part of the MedDiet, as well as a higher intake of monounsaturated fats (MUFA) in relation to saturated fats (SFA). Fourteen studies out of the 70 identified were using this definition to assess adherence to the MedDiet [7, 27, 41–52]. Nine other definitions, used in 18 studies, were relatively similar to this one (labelled from tMedDiet 1 to tMedDiet 9) [13, 53–68, 106]. The differences among these dietary pattern scores were observed in the cut-off values considered to define moderate alcohol intake as well as in how healthy fat intake was reflected. Regarding this last point two of these definitions, tMedDiet 4 and 5 [13, 58–62], considered intake of olive oil instead of the MUFA: SFA ratio. This modification has been commonly used in those studies conducted in non-Mediterranean countries, where the intake of MUFA could be mainly represented by the fat intake from meat rather than olive oil. Another variation of this definition, tMedDiet 1 [53–55], included the intake of polyunsaturated fatty acids (PUFA) together with MUFA.

**Table 2:** Description of the different scores identified representing MedDiet

	Vegetables	Legumes	Fruits/nuts	Cereals	Fish	Meat	Dairy products	Alcohol	Fat intake	Extras	Cited
MedDiet	1	1	2	3	–	1	1	n.d.	1	–	[2]
tMedDiet	1	1	1	1	1	1	1	1	1	–	[7, 27, 41–52]
tMedDiet 1	1	1	1	1	1	1	1	1	4	–	[53–55]
tMedDiet 2	1	1	2	1	1	1	1	3	1	–	[56]
tMedDiet 3	1	1	1	1	1	1	1	4	1	–	[57]
tMedDiet 4	1	1	1	1	1	1	1	1	2	–	[13, 58–61]
tMedDiet 5	1	1	1	1	1	1	1	–	2	–	[62]
tMedDiet 6	1	1	2	1	1	1	1	16	1	–	[63–65]
tMedDiet 7	1	1	2	1	1	1	1	14	1	–	[66]
tMedDiet 8	1	1	2	1	1	1	1	17	1	–	[67, 106]
tMedDiet 9	1	1	2	1	1	1	1	1	1		[68]
aMedDiet	1	1	2, 3	2	1	2	–	5	1	–	[69–77]
aMedDiet 1	1	1	2, 3	2	1	2	–	15	1	–	[78]
aMedDiet 2	1	1	2, 3	2	1	2	–	6	1	–	[79]
aMedDiet 3	1	1	2, 3	2	1	2	–	7	1	–	[80, 81]
aMedDiet 4	1	1	2, 3	2	1	2	2	6	1	–	[82]
MedDiet 1	2, 3	–	2	2	1	–	–	–	2	–	[83]
MedDiet 2	1, 4	–	2	2	1	3	3	8	3	–	[84]
MedDiet 3	1	2	2	1	1	1	1	13	4	–	[85, 86]
MedDiet 4	5*	*	2	3	1	1	1	1	6	–	[87, 88]
MedDiet 5	1	–	2	1	1	4	1	2	4	–	[89]
MedDiet 6	1	2	2	2	1	2, 5	2	14	5	–	[90]
MedDiet 7	8**	2	**	2	1	2	11	5	11	–	[91, 92]
MedDiet 8	1, 8	2, 3	1	2	1	3, 6, 9, 10	3	–	2	2, 5-10	[93]
MedDiet 9	1	–	2, 3	9	1	2, 3	12	5	1	4	[94]
MedDiet 10	1	1	2	2	1	2, 5	2	18	2	3	[95]
mMedDiet	6	3	–	1	1	1	1	–	1	–	[96]
iMedDiet	7	1	2	4	1	6	4	9	2	1, 2	[41, 97]
CA	1	–	2	5, 6	–	–	5, 6	8, 10	–	–	[98]
FA	1	–	–	4, 7	1	–	–	–	7	3	[99]
rmMedDiet	6	–	4	2	1	1	1	2	4	–	[100]
AHEI	1	2	5	2	–	2	–	11	8–10	2, 4	[101, 102]
sMedDiet	1	1	1	2	1	2, 5, 7	2, 7	12	2	3	[103, 104]
MAI	1	1	2	1	1	1, 7	8–10	8	2	2, 3, 5	[105]

Vegetables (+); 1: vegetables; 2: raw vegetables; 3: cooked vegetables; 4; salad; 5: vegetables + legumes; 6: vegetables + potatoes; 7: Mediterranean vegetables (raw tomatoes, leafy vegetables, onion and garlic, salad, fruiting vegetables); 8: vegetables + fruits (excl. potatoes and fruit juices)

Legumes (+); 1: legumes; 2; legumes + nuts; 3: legumes + nuts + seeds

Fruits/nuts (+); 1: fruits + nuts; 2: fruits; 3: nuts; 4; fruits + juices; 5: fresh fruit only

Cereals (+); 1: cereals; 2: whole grain cereals; 3; cereals + potatoes; 4: pasta; 5: whole grain bread; 6: pasta + rice; 7: bread; 8: refined cereals (−); 9: max score = 3rd quintile of intake (bread, rice and white potatoes)

Fish (+); 1: fish (and seafood)

Meat; 1: meat and meat products (−); 2: red and processed meat (−); 3: white meat (+); 4: meat, meat products and egg (−); 5: poultry (−); 6: red meat (−); 7: eggs (−); 8: preferred white meat over red meat and processed meat; 9: organ meat (−); 10: egg (+)

Dairy products; 1: dairy products (−); 2: high-fat dairy products (−); 3: dairy products (+); 4: butter (−); 5: full-cream milk (−); 6: butter (+); 7: low-fat dairy products (+); 8: Butter, margarine or cream; 9: milk; 10: cheese; 11: fermented dairy products (+); 12: max. score 3rd quintile of intake

Alcohol; 1: max. score = women 5–25 g/d, men 10–50 g/d; 2: max. score ≥ sex-specific median; 3: max. score > 0 drinks/wk ≤ 2 drinks/d; 4: max. score = women ≤ 1 drink/d, men ≤ 2 drinks/d; 5: max. score = women 5–15 g/d, men 10–25 g/d; 6: max score = 5–15 g/d; 7: max. score = women 5–15 g/d, men 10–15 g/d; 8: wine; 9: max. score ≥ 0–12 g/d; 10: beer (CA); 11: max. score = women 0.5–1.5 drinks/d, men 0.5–2.0 drinks/d; 12: max. socre < 3 glasses/d = 5 points and min. score > 7 glasses/d or none = 0 points; 13: max. score ≥ 1 drink/month; 14: max. score to those in the second quintile of alcohol intake; 15: max. score = 5–25 g/day for everybody; 16: max. sore = > 0–30 g/d; 17: max. score = women 1–7 drinks/wk, men 1–14 drinks/wk; 18: max. score 1–300 ml/d

Fats; 1: MUFA:SFA ratio; 2: Olive oil; 3: MUFA; 4: (MUFA + PUFA):SFA ratio; 5: PUFA + MUFA; 6: PUFA:SFA ratio; 7: Oils; 8: trans fatty acids; 9: EPA + DHA fatty acids; 10: PUFA; 11: olive oil and/or rapeseed oil as main sources of fat

Extras; 1: potatoes (-); 2; sugar-sweetened beverages (−); 3: potatoes (+); 4: Sodium (-); 5: confectionary; 6: fruit juices and drinks; 7: pickled food; 8: deep fried food; 9; salty snacks; 10: pizza

CA, cluster analysis; FA, factor analysis; AHEI, Alternative Healthy Eating Index; MAI, Mediterranean Adequacy Index; (−), not in line with the MedD; (+), in line with the MedD; MUFA, monosaturated fatty acids; SFA, saturated fatty acids; PUFA, polyunsaturated fatty acids; EPA, eicosapentaenoic acid; DHA, docosahexaenoic acid; n.d., not defined

The second main definition observed referred to the one created by Fung et al. [71], the alternative MedDiet index (aMedDiet). The authors modified the original score created by Trichopoulou et al. by considering some eating behaviours that were associated with lower risk of chronic disease. Thus, the authors separated into two different groups fruits and nuts, eliminated the dairy group, included whole grain products only, as well as only red and processed meat. Nine studies used this definition and five other studies used a definition relatively similar to this one [69–77]. The main difference was observed in how alcohol intake in moderation was defined, and also one of these definitions, aMedDiet 4 [82], included the food group high fat-dairy products as not being in line with the MedDiet.

Apart from these two main groups of definitions we identified 16 other scores used to assess MedDiet adherence in 24 reports [41, 83–105]. A great variability was observed among these different definitions (see Table 2). In addition, some of the meta-analyses here evaluated included also studies in which MedDiet adherence specifically was not intended to be assessed. This is for example the case of the meta-analyses from Bloomfield et al. [36] evaluating the effects of the MedDiet on cancer mortality and incidence. There, the authors also included two reports in which the Alternative Healthy Eating Index (AHEI) was assessed and not the MedDiet [101, 102]. In their meta-analysis on cancer mortality Bloomfield et al. also included the study of Menotti et al. [99], in which two dietary patterns were identified by means of factor analysis. Particularly, the authors observed a dietary pattern characterized by high consumption of bread, pasta, potatoes, vegetables, fish, and oil and by lower consumption of milk, sugar, fruit, and alcoholic beverages and argued this was similar to the MedDiet dietary pattern. Similarly, two of the meta-analyses here identified evaluating the effects of MedDiet on the onset of T2D [30, 40] included the study of Brunner et al. [98], in which dietary patterns were obtained by cluster analysis. In this study one of the clusters identified was strong and positively correlated with the intake of fruit, vegetables, rice, pasta, and wine, and thus named *Mediterranean*-*like* cluster.

### Test for subgroup differences comparing different types of MedDiet adherence scores

Several of the included meta-analyses reported moderate to high statistical heterogeneity, which could be related to the different indices applied. Once we identified the different scores used in the primary studies and considered these as similar enough, if at least two cohort studies were included in a meta-analysis for at least two different MedDiet scores, we combined the studies applying similar scores in a new meta-analysis and tested for possible subgroup differences in between the different scores.

Comparing the tMedDiet versus the aMedDiet score suggested no evidence for subgroup differences (*p* > 0.10) for T2D as outcome in the random effects model (Table 1). In the meta-analysis by Bloomfield et al. on cancer mortality, an inverse association was observed with aMedDiet (RR 0.81, 95% CI 0.78, 0.85), while no association with the tMedDiet was found (RR 0.92, 95% CI 0.64, 1.31). However, as in the re-analysis of the work from Sofi et al. for cancer mortality/incidence, no evidence for subgroup differences were observed in the study from Bloomfield et al. Regarding CVD, a marginal difference in the subgrouping (test for subgroup difference *p* = 0.06) was observed when reanalysing the meta-analysis by Sofi et al. [19], where the tMedDiet showed a stronger inverse association (RR 0.87, 95% CI 0.83, 0.91) compared to studies using the aMedDiet score (RR 0.92, 95% CI 0.89, 0.94). Nevertheless, no statistically significant subgroup differences were observed in any of the other meta-analyses. The fixed effects sensitivity analyses confirmed mainly the results of the random effects meta-analysis.

Unfortunately, in the case of the meta-analyses on cognitive-related diseases it was not possible to test for sub-group differences due to the small number of studies included.

## Discussion

In this umbrella review of meta-analyses, we summarized the findings from prospective cohort studies and investigated the different scores used to assess MedDiet and their implications on the risk of major chronic diseases (T2D, CVD, cancer and cognitive-related diseases). We observed that a higher adherence to the MedDiet was associated with lower incidence of T2D, lower incidence/mortality of CVD, and lower incidence/mortality of cancer; the credibility of this evidence ranged from low to moderate. Low credibility of the evidence implies that the confidence in the effect estimate was low and that further research will provide important evidence on the confidence and likely change the effect estimate, while moderate credibility means that further research could add evidence on the confidence and may change the effect estimate [26]. Two scores assessing adherence to the MedDiet were manly applied in cohort studies (tMedDiet and aMedDiet). Overall, we observed little difference in risk associations comparing tMedDiet versus aMedDiet indices in the subgroup meta-analysis. In the meta-analysis by Sofi et al. [19], which assessed the effect of the MedDiet on the risk of CVD incidence/mortality, some differences were observed; in this case, both MedDiet scores associated with lower risk, but the effects observed for the tMedDiet score were stronger.

### Mediterranean diet scores and health associations

Within the large variety of indices attempting to reflect adherence to the MedDiet two scores could be identified which were applied more frequently; the tMedDiet used in 32 studies [7, 13, 27, 41–68, 106], and the aMedDiet used in 14 studies [69–82]. Nineteen other definitions were found within the remaining 24 studies [41, 83–105]. These were very disperse; from dietary patterns derived by exploratory methods [98, 99] to other scores not explicitly created to assess MedDiet, such as the Alternative Healthy Eating Index [101, 102].

The tMedDiet score was used for the first time by Trichopoulou et al. [27] in the EPIC-Greece cohort in 2003, while the aMedDiet score was created and used for the first time by Fung et al. [70] in the Nurses’ Health Study. This second one was a literature-updated version according to published evidence. As stated before, for most of the analyses here conducted, similar associations were observed when applying one or the other score. In the meta-analysis by Bloomfield et al. differences in the estimates were observed but the test for subgroup differences was not significant. These observed differences could be due to the selection of healthier items included in the aMedDiet score compared to the tMedDiet. This is the case, for example, of whole grains; the most recent meta-analyses observed an inverse dose–response association of whole grains with cancer mortality [107, 108]. The evidence regarding the health implications of dairy products is controversial. A recent meta-analysis observed in a non-linear dose–response model that low intake of total dairy products could be protective against cancer-related deaths [109]. Concerning meat and processed meat, the World Cancer Research Fund International recommends an average consumption under 300 g a week, and to limit as much as possible the intake of processed meat [110]. The same report stated that processed meat has been particularly associated with an increased risk of colorectal and stomach non-cardia cancers. Finally, the inclusion of two food groups for fruits and nuts instead of combining them into one attributes a higher weight of these food items presumed to have a beneficial effect on health. These two food groups, as well as whole grains, are rich in fibre, nutrient particularly associated with lower risk of colorectal cancer [111]. In any case, our analyses are restricted to overall cancer mortality; the broad consideration of overall cancer and this approach could complicate possible conclusions and interpretations due to the different nature and aetiology of the different cancer sites. Still, no difference has been observed for T2D and CVD even though foods like whole grains and red meat have been also shown to be associated with these diseases [112–116].

Some other limitations should be mentioned. In the first place, our analyses have been restricted to the ones already performed by the authors and this could complicate the comparisons. For example, some of the authors combined incidence and mortality in one outcome while other preferred to assess these separately. Moreover, the definitions for the scores here identified were restricted to the ones previously identified by the authors conducting the meta-analyses we have here evaluated. Thus, other scores used to assess adherence to the MedDiet could not have been identified. This is the case, for example of the Mediterranean diet pyramid [117]. On the other hand, other sources of heterogeneity due to the construction of the scores have not been evaluated here. Moreover, umbrella reviews are limited by their primary objective. In our case we targeted meta-analyses on prospective observational studies and thus, no randomized controlled trials were included. Also, only studies included within the identified meta-analyses have been here evaluated; any other potentially relevant study could not have been included. A part from this, other interesting and possible sources of differences on the credibility and the heterogeneity observed in the meta-analyses (e.g. country affiliations of authors, year of publication) has not been evaluated within this work. For example, difference in the number of cohort studies included in the meta-analyses for a certain endpoint can be observed; this is due to the inclusion criteria restriction, some meta-analyses only included studies with healthy participant at baselines, like Jannasch et al. [16] while other did not consider this [28].

## Conclusion

In summary, most included meta-analyses reported an inverse association between high adherence to MedDiet and risk of chronic disease; however, the credibility of evidence was rated low to moderate. The present umbrella review shows considerable heterogeneity in the assessment of adherence to the MedDiet, which limits the comparability among primary studies. Two main scores [Trichopoulou MedDiet (tMedDiet) and alternative MedDiet (aMedDiet)] have been identified as the most used and we encourage researchers to use one of these two definitions when assessing adherence to the MedDiet if possible in order to not compromise further comparability among studies. The use of other scores would be justified in case these would better reflect the MedDiet for the purpose of the research. For most of the outcomes here evaluated we did not observe major differences in the use of one or the other of these two scores, nevertheless, some differences were observed for cancer mortality, fact that could be due to the betterment of the aMedDiet with regard to the tMedDiet according to the literature evidence.
